# Anxiety, Stress Perception, and Coping Strategies among Students with COVID-19 Exposure

**DOI:** 10.3390/jcm12134404

**Published:** 2023-06-30

**Authors:** Andrei Shpakou, Elżbieta Krajewska-Kułak, Mateusz Cybulski, Dorota Sokołowska, Małgorzata Andryszczyk, Ewa Kleszczewska, Yelena Loginovich, Jakub Owoc, Andrei Tarasov, Natalia Skoblina, Krystyna Kowalczuk

**Affiliations:** 1 Department of Integrated Medical Care, Faculty of Health Sciences, Medical University of Bialystok, 15-089 Bialystok, Poland; elzbieta.krajewska-kulak@umb.edu.pl (E.K.-K.); mateusz.cybulski@umb.edu.pl (M.C.); krystyna.kowalczuk@umb.edu.pl (K.K.); 2 Department of Physical Education, Faculty of Physical Education and Tourism, Eastern European University of Applied Sciences in Bialystok, 15-472 Bialystok, Poland; dorota.sokolowska@wsfiz.edu.pl; 3 Department of Health Care, Prof. Edward F. Szczepanik State Vocational College, 16-402 Suwałki, Poland; malgorzata.a@onet.eu (M.A.); kleszczewska.ewa@gmail.com (E.K.); 4 Department of Biology System and Genetic Research, Faculty of Animal Sciences, Lithuanian University of Health Sciences, 44307 Kaunas, Lithuania; lenalog77@gmail.com; 5 Department of Gerontology, Public Health and Didactics, National Institute of Geriatrics, Rheumatology and Rehabilitation, 02-637 Warsaw, Poland; 6 Department of Pediatrics and Preventive Medicine, Medical Institute, Immanuel Kant Baltic Federal University, 236041 Kaliningrad, Russia; drup1@yandex.ru; 7 Department of Hygiene, Pirogov Russian National Research Medical University, 117997 Moscow, Russia; skoblina_dom@mail.ru

**Keywords:** university students, anxiety stress perception, coping strategies, pandemic, COVID-19 patients, Poland, Lithuania, Russian exclave of Kaliningrad

## Abstract

**Background**: Studying anxiety, stress, and coping strategies during the COVID-19 pandemic is crucial to mitigate the negative effects associated with infection risk and disease consequences. **Objective**: This study aimed to investigate anxiety levels, stress perception, and coping strategies in relation to the presence of illness. **Material and Methods**: A cross-sectional online survey was conducted anonymously among 3950 university students from Poland (1822), Lithuania (232), and the Russian exclave of Kaliningrad (1896). Due to the nearly identical application of anti-epidemic measures, the respondents were treated as a unified group. The State-trait Anxiety Inventory (STAI), Perceived Stress Scale 10 (PSS-10), and mini-COPE scale questionnaires were used. Statistical analysis included the Shapiro–Wilk test to check normality, the Mann–Whitney U test for comparative analysis between groups, the Pearson χ^2^ test for categorical data, and Spearman coefficients for correlations between variables. **Results**: A significant proportion of young adults in the community exhibited symptoms of anxiety and stress during the COVID-19 pandemic. Among the 1212 men and 2738 women surveyed, 348 (28.7%) and 1020 (37.3%) individuals, respectively, were diagnosed with COVID-19 according to clinical protocols. Prolonged disease duration and more severe residual symptoms correlated with higher self-reported anxiety levels. **Conclusions**: The level of anxiety and stress varied depending on the duration of the disease, significantly impacting the choice of coping strategies. Overall, students displayed a proactive approach to coping activities but tended to postpone important decisions. Seeking social support was a prevalent coping mechanism, although respondents who had COVID-19 showed higher levels of concern for their own emotions, a tendency to discharge emotions through alcohol or other substances (male), and a greater reliance on religion (female). The study provides data that may be useful in developing educational and health policies focused on the mental well-being of university students and potentially other social groups.

## 1. Introduction

COVID-19 is a disease caused by the SARS-CoV-2 coronavirus. It is characterized by symptoms such as fever, cough, fatigue, loss of sense of smell and taste, and, in more severe cases, respiratory distress syndrome, which primarily affects adults [1]. While the severe form of COVID-19 is less common in young adults [2], the disease’s spread among this demographic carries significant social significance due to its high infectiousness and associated morbidity and mortality [3].

Contemporary research on the COVID-19 pandemic has focused on the emerging situation and the experiences of uncertainty, the threat of infection, disease symptoms, self-isolation, and quarantine. Individuals have approached this situation as a crisis with high stress potential [4]. Moreover, COVID-19 has been identified as an unexpected, large-scale event that disrupted community functioning and caused psychological trauma, as is evident from the literature [5]. Despite considerable efforts to control the situation, the virus continues to persist in many countries, with varying degrees of clinical manifestations [6].

The topic of mental health continues to be a prominent issue in medical care and public health, particularly in the context of ongoing pandemic waves. The universality of psychological reactions in humans during a pandemic can be understood by drawing analogies with reactions observed in other highly stressful situations [7,8]. The proliferation of the disease has created an environment where multiple factors simultaneously impact mental health indicators: (1) n unprecedented and potentially life-threatening situation of indefinite duration; (2) widespread restrictive measures that reduce the “psychological flexibility of the population” (insert citation for the quote); (3) the possibility of asymptomatic virus transmission and an increase in the number of mutations; (4) an unstable information landscape characterized by conflicting and abundant information on the subject; (5) uncertainty surrounding COVID-19 treatment; and (6) an unresolved vaccination situation [9].

Due to the widespread prevalence of coronavirus infection and its high neurotoxicity, even individuals who have never experienced mental health changes before are at risk [10]. The COVID-19 pandemic has led to a parallel epidemic of anxiety and depressive disorders, both during the disease and in the recovery phase [11]. The current stress situation, its significance, and the overall magnitude of post-COVID consequences necessitate changes in people’s daily lives and can result in a cumulative stress effect [12,13].

Recovered patients, especially ones who experienced severe COVID-19, face an increased risk of developing (post-COVID) post-traumatic stress disorder (PTSD) [14,15]. Patients with an optimistic outlook on life tend to have faster recovery rates compared to those with a pessimistic perception of their condition and surroundings [16]. Emotions, particularly anxiety, play a significant role in functioning, starting from the moment of exposure, through the onset of symptoms, the course of the disease, and even during the rehabilitation period. These circumstances influence individuals’ resilience to stress in threatening situations and can lead to alterations in their repertoire of coping strategies, resulting in stereotypical behaviors [17].

Considering the continuity of COVID-19 stages, its magnitude, variable course, and the vulnerability of young adult university students, especially women, it is crucial to study the characteristics of coping strategies. This research is essential to ensure appropriate and necessary psychological support for individuals who are infected, those in conditions with potential for infection, and those in the recovery phase [18,19]. It is widely recognized that the health, well-being, and social mood of students reflect the level of wellness, social stability, and overall life satisfaction within society as a whole [20].

A comparative stress-coping study conducted on identical populations in closely located cities within neighboring countries holds great promise for selecting optimal public health measures for the affected population [21]. The Kaliningrad region of Russia, as well as Lithuania and Poland, serve as suitable comparators due to their shared European Union (EU) border, as well as common histories, cultures, and religions. While these regions have more similarities than differences, they also exhibit some key distinctions. Notably, two of these countries are part of the EU. This study also allows for a comparison of the situation in the event of a COVID-19 crisis, considering the implementation of fairly restrictive anti-pandemic measures similar to lockdowns in these countries [22].

However, two neighboring countries on the EU’s eastern border, Belarus and Ukraine, were not included in this study. Belarus was excluded due to the different, often controversial, and more lenient anti-pandemic measures implemented by its government. Ukraine was not included in the comparison group due to the ongoing armed conflict with Russia, which has had distinct effects on the mental health of the population. It is worth mentioning that the authors have access to data for these two countries, and a subset of data concerning Belarus was already published in 2022 [23]. Given the limited number of epidemiological studies on the COVID-19 pandemic in Eastern Europe, this paper aims to fill the knowledge gap regarding the unique conditions for studying the spread of COVID-19 and its potential consequences on the health of young adults [24].

The main message of this manuscript emphasizes that increased individual risk for an unknown disease, coupled with the utilization of often ineffective emotional or behavioral strategies, can lead to changes in coping mechanisms during stressful situations and a deterioration in psychosomatic health. Considering the magnitude of the COVID-19 pandemic and the heightened vulnerability of young people, it is crucial to examine anxiety levels, stress perceptions, and the choice of coping strategies as indicators of mental health. This examination is necessary to provide appropriate and essential psychological support for individuals who have recovered from COVID-19 and those who are at risk of infection. Prolonged poor stress management can result in emotional and psychosomatic effects, including physical, cognitive, and emotional exhaustion, as well as reduced learning performance.

The aim of this study was to investigate the level of anxiety, both as a state reflecting the current experience of the situation and as a personality trait, along with perceptions of stress and coping strategies among student adolescents who were exposed to COVID-19. The study focused on three countries in the region along the eastern border of the European Union, where almost identical anti-epidemic measures were implemented. The study sought to address the following questions: (1) What was the frequency, expression, and severity of symptoms within the target group? (2) What were the frequency and characteristics of mental health indicators, such as anxiety as a trait and as a mental state, and susceptibility to stress in relation to COVID-19? Are there gender-based differences evident? (3) What were the prevalent stress coping strategies among students, and how were the choices of different options related to susceptibility to illness and gender? (4).

## 2. Material and Methods

### 2.1. Study Design and Setting

This cross-sectional study was conducted as part of the international multi-center research project known as “The COVID-19 Coping Study of Students from East Europe (SEECoping-S)”. The study utilized a cross-sectional survey conducted during January–February 2022, a period when the Omicron variant of the virus was widespread, and there was a significant increase in its incidence.

The cross-sectional survey aimed to gather reported information on the prevalence of anxiety and perceived stress among students from these three countries. The survey was administered online, ensuring the necessary assurances of anonymity to encourage respondents to provide accurate data on sensitive issues, particularly in the realm of mental health. No data were missing since the online platform did not allow incomplete student questionnaires to be submitted.

### 2.2. Participants

The online survey was conducted among a total of 3950 respondents from three countries situated on both sides of the eastern border of the European Union. Specifically, the respondents included individuals from Poland (PL, *n* = 1822), Lithuania (LT, *n* = 232), and the Russian exclave of Kaliningrad (RU, *n* = 1896), which is located on the border between Poland and Lithuania. The sample selection followed a simple random sampling method with a predetermined sample size.

The inclusion criteria for participants were as follows: being a student, between 18 and 25 years of age, and possessing the ability to read one of the four languages used in the survey (Russian, Polish, Lithuanian, and English). Regarding COVID-19 disease, confirmation was based on the presence of pathognomonic symptoms. In light of the most common symptoms associated with coronavirus infection in humans, including fever, taste and olfactory disturbances, dry cough, heavy breathing, weakness, and fatigue, the presence of at least three of these symptoms in combination with a positive RT-PCR (real-time polymerase chain reaction) test classified the respondent as having COVID-19. Criteria for excluding respondents from the study included the presence of any pre-existing mental illness, a history of mental disorders, and the potential influence of medication use within the month prior to the study.

### 2.3. Measures

To assess the various constructs, we employed well-validated and established measures in the form of standardized self-assessment questionnaires. The participants were initially asked to provide information regarding their sociodemographic characteristics, such as age, gender, and country of residence. Additionally, they were asked to disclose their health situation, including whether they had received vaccination against COVID-19. Moreover, participants were inquired about any previous diagnoses of COVID-19 among themselves and their partner or immediate family. The survey methodology underwent a standardization process, which was achieved through consensus among members of the international scientific research team.

### 2.4. Study Questionnaires

The COVID-19 pandemic and its consequences were identified in the questionnaire as the main stressors affecting daily living. The psychometric properties of the version of the STAI, PSS-10, and Mini-COPE inventory are considered good [25]. Participants were asked to self-assess primarily anxiety, understood as a transient and situationally conditioned state of the individual and anxiety understood as a relatively fixed personality trait, using translated versions of the standardized State-trait Questionnaire Inventory (STAI) [26,27]. Anxiety was measured as a trait (A/T) referring to the overall level of anxiety, and as a state (A/S) operationally defined as anxiety experienced at a given time or situation. The punctuation was determined by four possible responses for each item: (a) not at all, (b) a little, (c) moderately, and (d) very much; and (1) almost never, (2) sometimes, (3) often, and (4) almost always, respectively. Each of the 40 statements (20 for anxiety as a trait and 20 as a state) had three responses assigned to assess the intensity of the respondent’s emotions. The score of each test was calculated by summing the scores of each answer and scoring according to normalized severity indices. The results were then converted into numerical values to allow quantitative evaluation from 20 to 80 points. High numerical values indicate high levels of anxiety. Scores on each scale that are ≥30 points indicate moderate, while scores ≥45 determines severe anxiety. Cronbach alpha internal consistency coefficient calculated in this study was α = 0.831 and yielded satisfactory results (PL = 0.911; LT = 0.865; RU = 0.818). 

Stress levels over the past month were then assessed using standardized language versions of the Perceived Stress Scale (PSS) 10 questionnaire [25,28,29,30,31]. The degree of subjective perception of the stressful situation (10 questions) was determined in 5 gradations. The overall score characterized the degree of perceived stress in a gradation from minimum to maximum. Herein, the higher the score, the greater the sense of stress. Cronbach alpha internal consistency coefficient calculated in this study was α = 0.708 (PL = 0.662; LT = 0.610; RU = 0.717).

The degree of preference for coping strategies was determined using COPE (the Coping Orientations to Problems Experienced) mini-questionnaire [25]. Coping (14 strategies) was assessed using a shortened version of the Brief-COPE—Mini-COPE (28 questions) recommended in 1997 [32,33]. The tool is used to assess typical ways of reacting to situations of severe stress [34]. The strategies are divided into 4 categories (integral strategies) and corresponding scales: active coping (active coping, planning, positive revaluation), helplessness (taking psychoactive substances, doing nothing, and self-accusation), seeking support (seeking emotional and instrumental support), and avoidance behaviours (dealing with other things, denial, and giving vent to one’s feelings). Such strategies as turning to religion, acceptance, and a sense of humor constitute separate categories. Coping levels among respondents ranged from 0 (no use of that specific coping strategy) to 3 (the most frequently applied one) for each strategy. All responses were grouped into four main strategic coping factors: active coping, helplessness, seeking support, and avoidance coping [25]. The original Brief-COPE inventory and its Polish, Russian, and Lithuanian versions of the Mini-COPE questionnaire have been thoroughly revised and have clear scoring guidelines [25,35,36]. Cronbach alpha internal consistency coefficient calculated in this study was α = 0.749 (PL = 0.854; LT = 0.780; RU = 0.893). 

### 2.5. Procedure 

The invitation to participate in the online survey (Google Forms) was distributed through targeted advertisements, including the e-learning platform (Moodle), Skype, Microsoft Teams, and university social networks. The proposed information resources were available to students and were widely used in teaching during the COVID-19 pandemic. 

The clinical questionnaire included questions about the severity of the disease and an assessment of the effects on selected indicators of respondents’ mental health. The structured questionnaire provided important information on the severity of COVID-19 [16]. With the help of a clinical questionnaire, two groups were separated for further study: COVID-19 survivors—1368 (34.6%) in varying degrees of severity, and those who did not have the disease (healthy)—2582 (65.4%). 

Due to the lack of significant differences between country of residence, the respondents were treated as a unified group. Before initiating the study, permission was obtained from the leadership of the universities participating in the study and an ethics committee to conduct an anonymous survey of students. All participants were informed about the objectives of the study, the methodology, and the anonymous and confidential nature of the questionnaire. Access to the questionnaire was granted only if they agreed to participate in the study. No data were missing, since the online platform did not allow for submitting incomplete students’ questionnaires. All participants provided informed consent prior to completing the survey online (via computer by clicking “yes” after reading the study aims, methods, and confidentiality statement). The research was carried out in accordance with the Declaration of Helsinki and Good Clinical Practice in research. General ethical permission to conduct the study was obtained from the Bioethical Review Board at the Medical University of Bialystok, Poland (document number: APK. 002. 1932. 2022).

### 2.6. Statistical Analysis

Statistical analyses were conducted using the STATISTICA software package ver. 13.0. To account for potential confounders, all analyses were adjusted for gender and countries a priori. Normality was assessed using the Shapiro–Wilk test, revealing that the distribution of the quantitative data deviated from the normal pattern. Consequently, both nonparametric and parametric statistical methods were employed.

For dependent variables that followed a normal distribution, the mean (M) and standard deviation (SD) were calculated. For non-normally distributed data, the median (Me) was computed. The t-test for independent samples was used for comparative analysis between the selected groups. Additionally, in cases with large SD values, the non-parametric Mann–Whitney U test was employed. Qualitative variables were analyzed using frequencies and percentages. Categorical data were compared using the Pearson χ^2^ test. Spearman correlation coefficients were calculated to determine the strength and direction of associations between variables. Statistical parameters were estimated at a 95% confidence interval, while significance tests and confidence intervals were calculated at a significance level of 0.05. For all analyses, *p*-values less than 0.05 were considered statistically significant.

## 3. Results

### 3.1. Characteristics of the Sample

All analyses were adjusted for age, gender, as these were considered to be potential limiting factors a priori. The study focused on students between the ages of 18 and 25 (22.6 ± 5.35). Disease survivors were slightly older: 23.1 ± 5.74 vs. 22.3 ± 5.11 (*p* < 0.05). The dominant part of the sample was women: 2738 (69.3%). The ratio of men to women in the study groups reflects the general trend in the ratio of men to women in university faculties in the three countries. Among 1212 men, 348 (28.7%), and among 2738 women, 1020 (37.3%) were diagnosed with COVID-19 according to the clinical protocol. Table 1 shows the characteristics of the respondent sample related to membership in the group of healthy and those who had the disease, broken down by gender.

### 3.2. Main Findings 

Our main set of analyses focused on a section asking about the various symptoms and feelings that people may experience with the developing disease. Clinical symptoms associated with SARS-CoV-2 infection and COVID-19 severity were analyzed. Of the 1368 students, 166 (12.1%) were virtually asymptomatic (only fatigue, headache or sore throat were noted), 478 (34.9%) were mild, 629 (46.0%) were moderate, and 95 (6.9%) were severe (hospitalized). Common symptoms included smell reduction, that is, the partial or complete loss of olfaction/reduction of smell sensation—916 (67.6%); fatigue—873 (63.8%); headache—814 (59.5%); taste reduction—799 (58.7%); wheeze—552 (40.4%); cough—530 (38.7%); rash—157 (11.5%); and diarrhea—123 (9.0%). Moreover, 710 people (51.9%) had pyrexia at >37.5 °C. Each of those affected (in addition to being asymptomatic) had a combination of 4 or more symptoms. The strength of the correlation between disease severity and individual symptoms (wheezing, cough, fatigue, headache, smell or taste reduction, and their combination) was average (*r* = 0.45–0.50, *p* < 0.05) or weak (*r* = 0.20–0.35, *p* < 0.05) (in case of fever). The correlation between the number of symptoms and disease severity was at a high level (*r* = 0.75, *p* < 0.01). As the severity of the disease increased, the importance of such symptoms as smell reduction, taste reduction, fever, wheezing, headache, fatigue, and cough or their combination increased.

### 3.3. Anxiety

The specific impact of belonging to the group of healthy or affected people was significantly reflected in an important mental health indicator of anxiety (trait and state), among others. Statistical results obtained for the overall mean according to the STAI questionnaire (trait anxiety) was 41.4 ± 12.2, and for state anxiety: −46.1 ± 10.6 (*p* < 0.05). The increase in the difference between trait and state anxiety levels at 4.7 ± 8.61 indicated that the trait was rooted and the process was chronic. The analysis of the surveys shows that with regard to disease severity, the level of anxiety proved to be a differentiating factor between the two groups.

Careful comparisons showed that the lowest anxiety levels as a state were declared by men who did not have COVID-19. A more detailed analysis found that the prevalence of high anxiety (trait) (>45 points) among unaffected students was 35.2%, with anxiety as a state being 50.6%. Students who had COVID-19 experienced higher levels of anxiety (trait and state) than respondents in the unaffected group. When considering the normal values for both genders, high levels of anxiety were found in both male and female groups. Nevertheless, women were more likely to have more severe anxiety symptoms. Basic descriptive values and comparisons of the intensity of anxiety related to the COVID-19 pandemic by groups and gender are presented in Table 2.

The prevalence of high levels of anxiety was higher among qualified patients (both women and men). The prevalence of high anxiety (trait) expressed as a percentage was higher in women at 38.5% than in men at 30.8% (*p* < 0.001). Similar results were obtained for anxiety (state) (58.4% vs. 39.3%).

### 3.4. The Perceived Stress Scale (PSS-10)

We obtained the stress distribution for our sample, as indicated in Table 3. Here, high stress corresponds to a score one *SD* above the mean. Low stress corresponds to a score one *SD* below the average.

The incidence of anxiety and stress was related to gender, especially in the convalescent group. COVID-19 patients hospitalized during the pandemic often suffered from psychological distress after hospital discharge. For a more detailed specification of stress-coping scenarios, it was necessary to rank the selected methods, which was achieved by assessing coping strategies. High rates prevailed among women. Subjective perceptions of the overall level of tension in a stressful situation allowed us to assess and account for efforts to counteract stress.

### 3.5. Coping Strategies, Measured by the Mini-COPE Scale

Respondents who were not affected by the disease showed a statistically significant moderate negative relationship between their level of using strategies focused on active coping and anxiety. Accordingly, the more intensely students dealt with stressful situations proactively, the less they experienced negative symptoms of anxiety. A statistically significant yet weak negative correlation was shown among convalescent respondents. The helplessness strategy demonstrated a moderately positive relationship with anxiety in both groups, but the strength of the relationship was more pronounced in those in the second group. Avoidance behaviour strategy correlated with anxiety, and the strength of the relationship was similar in both groups. A weak association characterizes avoidant behaviour with anxiety as a trait and a moderate association with anxiety as a state.

Respondents who underwent COVID-19 differed from healthy individuals in having higher levels of concern about their own emotions, and a tendency to discharge them (an integral factor of avoidance coping). They were more likely to postpone important decisions in the context of coping in an effort to avoid stress and were characterized by more activities oriented toward seeking social support. Furthermore, they were more likely to engage in passive coping strategies, with the extended duration of the pandemic and its severity increasing the frequency of use (Table 4).

Tactics for choosing coping strategies among non-afflicted and healthy individuals were similar by gender. In the context of active coping, the differences between men and women are minimal. Respondents in both groups used psychoactive substances at a similarly low rate (this rate increased among students who had COVID-19, especially women), as is the dominant turn to religion among women in this group. However, the repertoire of coping strategies among women was broader than among men due to the focus on emotions and the expression of feelings. The risk of possible infection and apparent disease often activated coping strategies related to active functioning. In summary, it is worth highlighting that a notable proportion of young adults experienced symptoms of anxiety and stress. Furthermore, increased duration of illness and the presence of more severe residual symptoms and sequelae were associated with higher self-reported levels of anxiety and stress among patients. The severity of the disease also influenced individuals’ coping strategies for managing stress. Specifically, respondents who had contracted COVID-19 exhibited differences compared to those who had not, such as higher levels of anxiety regarding their own emotions and a tendency to seek release through the use of alcohol or other psychoactive substances. Additionally, there was a stronger attachment to religious beliefs, particularly among women.

## 4. Discussion

The purpose of our study was to narrow down the possible correlates of anxiety and stress, as well as potential coping mechanisms among male and female students according to the presence of COVID-19 disease, from three countries along the eastern border of the European Union.

Young adult students were chosen as the study group, because the physical and mental health, as well as the social mood of this target group, reflects the level of well-being, social stability, and degree of satisfaction with life in the greater society [37]. As students are distinguished and characterized by distinctiveness, a sense of in-group community and a tightly organized group, and the strictness and disproportionality of the consequences of anti-pandemic measures have affected them greatly compared to other age and social groups [38]. Studies on the mental health of this target group were conducted in the very first weeks of the COVID-19 outbreak in China and found that the epidemic had a significant effect on the mental health of students, and those who were affected present symptoms of disorders similar to those who have suffered traumatic stress [39].

The selection of the study’s geographic area was based on its location on the eastern border of the EU, where several countries with closely situated university towns [40] have populations of young people that are nearly identical. Moreover, these countries implemented similar measures in response to the pandemic and there were minor differences in the population prevalence of COVID-19. Additionally, the region demonstrates a strong commitment to reform and an active pursuit of optimal public health measures [41]. The gender-specific characteristics of adolescent mental health are also an important aspect highlighted in the study [42]. This, too, must be taken into account when conducting a study noting the differences in mental health indicators in subgroups of men and women.

The results suggest two implications for student mental health indicators. The first refers to the negative effect of the COVID-19 situation on mental health, expressed in high levels of anxiety and stress. The second refers to the high adoption of active coping mechanisms. This is a dynamic process that changes as people interact with the environment, and can be stable or unstable at different stages of adaptation to new conditions. We confirmed the high adaptation rate among students during the COVID-19 pandemic. Adaptive coping is a protective factor for students’ mental health and can be viewed as a buffer that attenuates the negative impact of COVID-19-related stressors on perceptions concerning COVID-19 infection (or mental health) risk [42]. The pandemic and the severe restrictive measures imposed as a result have contributed to accepting the reality of what happened and assessing the timeliness of the problem. According to the study, anxiety and stress levels were high compared to results from individual countries [43], which showed that about a third of the adult population suffered from anxiety, and more than half from stress. The findings from the UK survey conducted within 20 weeks of the country’s quarantine announcement [44] suggest that anxiety reached the highest levels in the early stages of isolation, but declined subsequently, probably because people adjusted to the circumstances. According to our survey, the prevalence of high anxiety (trait) among unaffected students was 35.2%, with anxiety as a state reaching 50.6%. Students who underwent COVID-19 had even higher anxiety levels than respondents in the unaffected group. This is explained by the fact that respondents who were infected with coronavirus were actually frightened by their disease. Their anxiety levels were increased by the uncertain course of the disease and its consequences, forced isolation or hospitalization, and fear of death. Again, high anxiety rates recorded among the healthy indicated an increasing problem [45]. This can be attributed to their exposure to an information field that induces anxiety. It can be further compounded by the prevailing societal mood characterized by chronic uncertainty, economic changes, and dissatisfaction with the state’s healthcare efforts [46]. Researchers have observed that socioeconomic insecurity has contributed to an increase in mental disorders associated with the COVID-19 pandemic [47].

In contrast to early studies conducted before the COVID-19 era [48], our study provides a comprehensive assessment of outcomes during the extended duration of the pandemic, taking gender into consideration. The prevalence of high anxiety (trait) was found to be higher in women, with 38.5% compared to 30.8% in men (*p* < 0.001). Similar results were observed for anxiety (state), with rates of 58.4% in women and 39.3% in men. A study conducted in Turkey also confirmed that nearly half of the participants experienced anxiety, with a higher prevalence among women [49]. This may be attributed to the well-established connections between women’s emotional resilience and low self-esteem in stressful situations, which can contribute to a loss of balanced predisposition and self-control. Maintaining healthy levels of self-esteem is essential for effectively coping with anxiety. Our survey also shows a higher prevalence of stress. The perception of stress is a subjective and variable phenomenon. Special attention is paid to the processes of coping with stress, which determines the positive and negative effects of stress on the individual. A significant proportion of COVID-19 patients reported symptoms in situations of psychological distress. The course of coping was found to depend on personal resources, social support, attitudes toward the disease, and the severity of its symptoms [50]. In general, it can be concluded that the higher stress levels of students compared to data from the general population may be related to the commitment and challenges of their “working and studying,” which is consistent with previous reports [51]. It should be noted that our representative sample (students aged 18 to 25) had higher anxiety and stress levels compared to other age groups, as also reported by other authors [18]. The proportion of those affected with high levels of stress is comparable to proportions observed in recent studies [42].

Differences by gender are characteristic of two scales: total “overload” and “perception of stress”. The average stress level was 20.6 among the healthy and 21.7 among convalescents, which was higher than in the general population (13.02) [51]. The presence of a gender difference in stress among university students is also consistent with the current literature: most studies have reported that stress is higher in female students [52]. Based on this, it appears that women are more susceptible to experiencing the consequences of COVID-19 compared to men.

The COVID-19 pandemic not only affected the intensity of stress, but also changed and diversified coping strategies. Having effective strategies for stressful situations is important because they can prevent experiences that lead to mental disorders related to a critical situations [53]. People use different methods to cope with stress, as was observed in our study as well. Regardless of their attitudes toward the disease, the respondents focused on active ways of coping and positive reformulation, meaning: they chose to focus on the problem and seek instrumental support (i.e., seeking and receiving advice and help from others), as well as emotional support.

Coping is a complex construct that can play a significant role in protecting against or increasing the risk of adverse mental health outcomes during stressful life experiences [54]. No significant differences were found in the choice of active coping strategies in terms of gender, as reported by other researchers [42]. Quarantine measures, the severity of the disease, and often inadequate information about the epidemic situation influenced the coping strategies chosen: students who were not sick were more likely to choose active coping and planning, but often refused to believe what happened. Respondents who have undergone coronavirus infection differ in their actions in that they are more likely to choose an avoidance strategy and are less likely to plan. This is most likely due to the fact that the symptoms of asthenic syndrome include chronic and rapid fatigue. In addition, depressive moods, loss of energy, and reduced interests tend to dominate in both study groups. It is worth noting that convalescents exhibit maladaptive behavior due to distress, most likely caused by a lack of understanding of their future actions. A statistically significant difference was found for the strategy of self-distraction, meaning: engaging in other activities to avoid thinking about an unpleasant situation. This strategy was more common among convalescents. They accepted the reality, but more often turned to the use of “tranquilizers” (medications, alcohol) to cope with the situation.

Gender-related differences were also observed in coping strategies. Women were more likely to utilize emotion-focused coping strategies, emphasizing negative experiences and engaging in mental and behavioral withdrawal. The interaction between gender and health conditions influenced women’s typical coping behaviors—they sought emotional support as well as instrumental support, such as seeking advice, assistance, and information on managing difficulties.

Women who had no direct exposure to the disease found it more challenging to accept the situation and denied the reality of the pandemic. In contrast, men tended to be more proactive in distracting themselves from unpleasant thoughts and sought positive ways to cope, such as engaging in physical activity. However, men generally avoided seeking both instrumental and emotional social support, unlike women.

The outcome of coping is influenced by the individual’s engagement in the coping process, which serves as a means of self-realization and supports sustainable personal development. This outcome is linked to various factors, including the evaluation of the situation, the perception of self-realization possibilities within the given context, commitment to specific activities, and the subjective choice of appropriate or inappropriate coping strategies, which may pose developmental risks. Developing precise risk communication strategies that enhance risk assessment perception and self-efficacy can facilitate desirable and effective practices, not only for preventing COVID-19 but also for preventing other infections [55]. Our results stress the need to design prevention and intervention programs to reduce the negative consequences of COVID-19. There is a need to inform people about available resources and practical methods to deal with these emerging issues, along with the continuing stress of COVID-19. As COVID-19 disrupts communities around the world, further research and understanding of effective coping are crucial to reducing the short- and long-term impact of the pandemic on the psyche of young people [56].

## 5. Limitations

This study has some limitations that are typical for online surveys. The results are also limited to students and may not be applied to other groups or the general population. We collected data using self-reported questionnaires that are commonly used; however, they may not provide a complete picture of mental health. It is also important to note that professional and accurate assessment of mental disorders can only be done by professional psychologists or psychotherapists. The study’s findings do not represent the overall impact of COVID-19 on mental health, but they help identify areas in which students might need psychological assistance in critical situations. Finally, it is important to note that the study captures a relatively narrow time frame, which may not apply to other, possibly longer waves of the pandemic.

## 6. Conclusions

A large percentage of young adults in the community demonstrated anxiety and stress symptoms during the COVID-19 pandemic. The level of anxiety and stress varies according to the duration of the disease which significantly affects the choice of coping strategies. There was a reciprocal correlation between exposure to COVID-19 among students and anxiety, levels of perceived stress, and coping activities. For the most part, students were characterized by a clear level of active coping activities. However, they postponed important decisions in the context of coping and were characterized by a preponderance of activities oriented toward seeking social support. The presence and severity of the disease changed coping strategies. Respondents who had COVID-19 differed from those without the disease in having higher levels of concern about their own emotions and a tendency to discharge them, especially due to the use of alcohol or other psychoactive substances (male), as well as more frequently turning to religion (female).

Given the evolving nature of the COVID-19 pandemic, our study results may serve as a starting point for future cross-border research on the physical and mental health of diverse social groups. This study offers data that may be informative for developing educational and health policies focused on the mental well-being of university students. It is crucial for universities to collaborate with psychological services in order to monitor and address stress and anxiety associated with the pandemic. To promote psychological adaptation, students should have access to programs such as seminars and team-building activities. Additionally, implementing a COVID-19 disease scale management strategy is necessary. Public education initiatives should focus on coping strategies, effective disease prevention methods, and practical resources for assistance. This process should be regarded as a long-term endeavor that begins during the pandemic and continues even after its conclusion.

## Figures and Tables

**Table 1:** Characteristics of the study population by gender of healthy respondents and those who were infected, developing COVID-19 symptoms.

	Have Not Had a COVID-19 Infection (*n* = 2582)			Recovered from COVID-19 Infection (*n* = 1368)			Total (*n* = 3950)		
	Male (*n* = 864)	Female (*n* = 1718)	*t*-Test for Age	Male (*n* = 348)	Female (*n* = 1020)	*t*-Test for Age	Male (*n* = 1212)	Female (*n* = 2738)	*t*-Test for Age
Age, mean (years ± SD)	21.1 ± 3.91	22.9 ± 5.53	−8.20; *p* < 0.01	21.3 ± 4.22	23.7 ± 6.06	−6.79; *p* < 0.001	21.2 ± 4.0	23.2 ± 5.74	−10.94; *p* < 0.001
	22.3 ± 5.11			23.1 ± 5.74			22.6 ± 5.35		−4.50; *p* < 0.01
Vaccinated against COVID-19, *N*; % (95%CI)	605; 70.0; (67.0–73.0)	1251; 72.8; (70.1–74.9)	*χ*^2^ = 2.2; *p* > 0.05	235; 67.5; (61.3–71.1)	741; 72.6; (70.1–75.5)	*χ*^2^ = 3.32; *p >* 0.05	840; 69.3; (66.3–71.5)	1992; 72.8; (71.2–74.4)	*χ*^2^ = 4.92; *p* < 0.05
	1856; 71.9; (70.1–73.6)			976; 71.3; (68.8–73.5)			2832; 71.7; (70.2–73.0)		*χ*^2^ = 0.7; *p* > 0.05
Contact with persons who have been diagnosed with COVID-19	534; 61.8; (59.0–65.4)	1100; 64.0; (62.3–66.8)	*χ*^2^ = 1.2; *p* > 0.05	295; 84.8; (81.2–88.6)	920; 90.2; (88.8–92.3)	*χ*^2^ = 7.7; *p* < 0.01	829; 68.4; (66.2–71.4)	2020; 73.8; (72.7–75.9)	*χ*^2^ = 12.1; *p* < 0.001
	1634; 63.3; (62.0–65.6)			1215; 88.8; (87.5–90.7)			2849; 72.1; (71.3–74.0)		*χ*^2^ = 289.9; *p* < 0.001

Note: *n* is the number of observations, % is the percentage of the total number of study participants in a given group; 95%CI—95% confidence interval; *SD*—standard deviation; *t*-test—a value of the Student’s *t*-test comparing healthy vs. those who had COVID; *χ*^2^—Pearson’s chi-squared test.

**Table 2:** The comparison of the trait anxiety and state anxiety scores and dependence on affliction state and gender of the respondents (*M* ± *SD*).

Variation in State Anxiety	Have Not Had COVID-19 Infection 1		Recovered from COVID-19 Infection 2		Total Sample		*t*-Test
	Male [M]	Female [F]	Male [M]	Female [F]	Male [M]	Female [F]	
Anxiety (trait)	38.3 ± 11.8	42.1 ± 11.9 *	40.8 ± 12.1	43.0 ± 12.5 *	39.0 ± 12.0	42.4 ± 12.1 *	*p*_[M1–M2]_ < 0.01 *p*_[F1–F2]_ < 0.01
	40.8 ± 12.0		42.4 ± 12.4		41.4 ± 12.2		*p*_[1–2]_ < 0.001
Anxiety (state)	41.9 ± 10.6	48.4 ± 10.2 *	44.2 ± 10.6	48.2 ± 10.0 *	42.6 ± 10.6	47.7 ± 10.1 *	*p*_[M1–M2]_ < 0.01 *p*_[F1–F2]_ > 0.05
	45.6 ± 10.7		47.2 ± 10.3		41.4 ± 12.2		*p*_[1–2]_ < 0.001
Anxiety levels (trait) (*n*, %, 95%CI)							
Low (<30)	605; 23.4 (21.8–25.1)		250; 18.3; (16.2–20.3)		855; 21.6 (20.4–22.9)		χ^2^ = 14; *p*_[1–2]_ < 0.01
Moderate (30–45)	1069; 41.4 (39.5–43.3)		600; 43.9 (41.2–46.5)		1669; 42.3 (40.7–43.8)		N/S
High (>45)	908; 35.2 (33.3–37.0)		518; 37.9 (35.3–40.4)		1426; 36.1 (34.6–37.6)		*χ*^2^ = 7.1; *p*_[1–2]_ < 0.05
Anxiety levels (state) (*n*, %, 95%CI)							
Low (<30)	229; 8.9 (7.8–9.9)		89; 6.5 (5.2–7.8)		318; 8.1 (7.2–8.9)		*χ*^2^ = 13.4; *p*_[1–2]_ < 0.01
Moderate (30–45)	1046; 40.5 (38.6–42.4)		512; 37.4 (34.9–40.0)		1558; 39.4 (37.9–41.0)		*χ*^2^ = 3.2; *p*_[1–2]_ > 0.05
High (>45)	1307; 50.6 (48.7–52.6)		767; 56.1 (53.4–58.7)		2074; 52.5 (51.0–54.1)		*χ*^2^ = 18.2; *p*_[1–2]_ < 0.01

Note: * *t*-test—value of the Student’s *t*-test between male and female (*p* < 0.05).

**Table 3:** Distribution of the respondents according to the degree of stress as categorized by the normative data for the PSS-10 (N, %, 95%CI).

Variation in Stress	Have Not Had COVID-19 Infection 1		Recovered from COVID-19 Infection 2		Total Sample		χ^2^ for Group Comparison and Total M and F
	Male [M]	Female [F]	Male [M]	Female [F]	Male [M]	Female [F]	
Low stress (0–13)	105; 12.2 (10.0–14.3)	94; 5.5 (4.4–6.6) *	39; 11.2 (7.9–14.5)	58; 5.7 (4.3–7.1) *	144; 11.9 (10.1–13.7)	152; 5.6 (4.7–6.4)	*χ*^2^ = 45.3 *p*_M–F_ < 0.001
	199; 7.7 (6.7–8.7)		97; 7.1 (5.7–8.5)		296; 7.5 (6.7–8.3)		*χ*^2^ = 0.41 *p*_1–2_ N/S
Moderate (14–26)	675; 78.1 (75.4–80.9)	1291; 75.1 (73.1–77.2)	262; 75.3 (70.8–79.8)	744; 72.9 (70.2–75.7)	937; 77.3 (75.0–79.7)	2035; 74.3 (72.7–76.0)	*χ*^2^ = 4.0 *p*_M–F_ < 0.05
	1966; 76.1 (74.5–77.8)		1006; 73.5 (71.2–75.96)		2972; 75.2 (73.9–76.6)		*χ*^2^ = 3.16 *p*_1–2_ N/S
High stress (27–40)	84; 9.7 (7.7–11.7)	333; 19.4 (17.5–21.3) *	47; 13.5 (9.9–17.1)	218; 21.4 (18.9–23.9) *	131; 10.8 (9.1–12.6)	551; 20.1 (18.6–21.6)	*χ*^2^ = 32 *p*_M–F_ < 0.001
	417; 16.2 (14.7–17.6)		265; 19.4 (17.3–21.5)		682; 17.3 (16.1–18.5)		*χ*^2^ = 3.93, *p*_1–2_ < 0.05

Note: * test *χ*^2^ for group male and female (*p* < 0.05).

**Table 4:** Aspects of coping strategies among students in the surveyed groups with and without COVID-19 infection, taking into account gender.

Coping Scales and Integral Strategies	Have Not Had COVID-19 Infection			Recovered from COVID-19 Infection		
	Male	Female	Test *U* Mann–Whitney [M] vs. [F]	Male	Female	Test *U* Mann–Whitney [M] vs. [F]
Active	2.07 ± 0.73	2.08 ± 0.67	739,816	2.09 ± 0.78	2.09 ± 0.70	172,933
Planning	1.96 ± 0.78	1.98 ± 0.70	740,532	1.95 ± 0.80	1.99 ± 0.69	177,406
Positive reframing	1.64 ± 0.82	1.69 ± 0.79	717,432	1.67 ± 0.82	1.70 ± 0.81	171,384
Acceptance	1.67 ± 0.80	1.73 ± 0.70	713,925	1.75 ± 0.77	1.76 ± 0.73	176,688
Humour	1.51 ± 0.90	1.27 ± 0.89	628,825 *	1.66 ± 0.92 ^#^	1.32 ± 0.92	139,611 *
Religion	0.60 ± 0.85	0.79 ± 0.91	647,150 *	0.59 ± 0.82	0.99 ± 0.97 ^##^	145,279 *
Use of emotional support	1.63 ± 0.87	1.95 ± 0.81	584,460 *	1.67 ± 0.87	1.94 ± 0.78	146,804 *
Use of instrumental support	1.49 ± 0.82	1.82 ± 0.78	574,908 *	1.50 ± 0.88	1.80 ± 0.78	142,780 *
Self-distraction	0.98 ± 0.67	1.08 ± 0.65	673,400 *	1.01 ± 0.68	1.11 ± 0.66	159,896 *
Denial	0.57 ± 0.69	0.73 ± 0.71	637,077 *	0.64 ± 0.74	0.77 ± 0.75	158,052 *
Venting	1.19 ± 0.71	1.45 ± 0.70	584,272 *	1.32 ± 0.73 ^#^	1.56 ± 0.72	154,113 *
Substance use	0.39 ± 0.70	0.36 ± 0.64	732,727	0.52 ± 0.70	0.58 ± 0.69 ^##^	174,949
Behavioural disengagement	0.58 ± 0.65	0.68 ± 0.63	663,297 *	0.61 ± 0.67	0.70 ± 0.66	162,232 *
Self-blame	1.24 ± 0.86	1.24 ± 0.87	740,269	1.29 ± 0.89	1.30 ± 0.90	175,504
Integral strategies						
Active coping	1.89 ± 0.64	1.92 ± 0.58	734,846	1.90 ± 0.65	1.93 ± 0.59	176,949
Helplessness	0.74 ± 0.56	0.76 ± 0.55	720,912	0.77 ± 0.57	0.81 ± 0.56	168,992
Seeking support	1.56 ± 0.79	1.89 ± 0.73	563,814 *	1.58 ± 0.82	1.87 ± 0.72	141,908 *
Avoidance coping	0.91 ± 0.54	1.09 ± 0.5 ^##^	589,235 *	0.99 ± 0.57	1.12 ± 0.53	150,008 *

Note: *M*—mean value; *SD*—standard deviation; * differences between males and females in the group (*p* < 0.05); ^#^ differences between males between groups; ^##^ differences between females between groups (*p* < 0.05).

## Data Availability

The data that support the findings of this study are available on request from the corresponding author. The data are not publicly available due to privacy restrictions.
